# Supplementary material for: Targeting the Id1-Kif11 Axis in Triple-Negative Breast Cancer Using Combination Therapy
Source: Biomolecules. 2020 Sep 8;10(9):1295. doi: 10.3390/biom10091295 (PMC7565337; doi:10.3390/biom10091295)
Supplement: Supplementary file 1 [file biomolecules-10-01295-s001.zip › supplementary files/Supplementary Information 31082020.docx]

**Supplementary Information**

**Supplementary Figure 1. Identification of putative *Id* regulated genes**

(a) The gene expression profile of three independent replicates of *Id* KD (with and without Doxycycline treatment), were compared by microarray analysis to generate pathway analysis of differentially expressed genes involved in different processes like cell cycle between Id depleted and Control 4T1 cells. (b) The gene expression profiles of the Id+ and Id- cells from three independent Id1C3Tag tumours were compared by RNA sequencing and analysis of differentially expressed genes between the Id+ and Id- mouse TNBC tumour cells were obtained which was correlated to pathways like cancer. (c) Aiming to discover high confidence genes involved in the *Id* gene regulatory network, lists of differentially expressed genes between the Id1 expressing or *Id* depleted TNBC models and their controls were compared using MetaCoreTM software. By comparing these two datasets, lists of MetaCore network objects common to both experiments as well as those unique to each of the two data sets was generated. [Results](https://www.ncbi.nlm.nih.gov/pmc/articles/PMC3531499/#s2) are visualized using the enrichment map plug-in for Cytoscape. Each circular node is a gene set with diameter proportional to the number of genes. The outer node color represents the magnitude and direction of enrichment in Id1C3Tag cells, inner node color enrichment in *Id* KD cells. Thickness of the edges (green lines) is proportional to the similarity of gene sets between linked nodes. The most related clusters are placed nearest to each other. The functions of prominent clusters are shown.

**Supplementary Figure 2. Effect of *Id* knockdown on EMT markers and self- renewal**

Relative protein expression level (a) and quantification (b) of E-cadherin and Vimentin in Id KD cells with respect to control cells were quantified with western blot. *β-actin* was used as the loading control. (c) EMT score calculated for Control and *Id* KD samples on a scale of 0 (fully epithelial) to 2 (fully mesenchymal). (d) Representative images and quantification of primary spheres under Control and Id KD conditions.

**Supplementary Figure 3. *Kif11* and *Aurka* are *Id* targets**

(a) There was a significant decrease in cell viability under *Kif11* KD and *Aurka* KD when compared to Control. (b) Phase contrast images and cell viability of 4T1 cells under Control, *Ccnd1*KD and *Casc5* KD. (c) Relative mRNA expression level of *Ccnd1* and Casc5 under Ccnd1 and *Casc5* KD condition compared to the Control. (d) Immunofluorescence images showing DAPI staining of Control, *Id* KD, *Kif11* KD and *Aurka* KD cells under fluorescence microscope. Representative images are taken using Nicon A1R+ confocal system at 100x magnification with scale bar corresponds to 100um, inset shows the 100x zoomed images of the same. All experiments were performed in three biological replicates and data were expressed as mean ± standard deviation. Unpaired Student’s t-test, two- way ANOVA. *p<0.05, **p<0.01, ***p<0.001, ****p<0.0001.

**Supplementary Figure 4. Determining the IC50 values for chemotherapy and small molecule inhibitors.**

(a) IC50 values for two commonly used chemotherapy drugs in breast cancer treatment, Paclitaxel and Doxorubicin; and the small molecule inhibitors of Kif11 and Aurka, Ispinesib and Alisertib respectively were determined. (b) Relative protein expression of Id1, Kif11 and Aurka in Paclitaxel, Ispinesib and Paclitaxel+Ispinesib treated 4T1 cells. (c, d) Percentage cells expressing Id1 and Kif11 in Control, Paclitaxel, Doxorubicin, Ispinesib and Alisertib treated cells). (e) KM plotter analysis of relapse free-survival of TNBC patients with *ID1* and *KIF11* gene expression. All experiments were performed in three biological replicates and data were expressed as mean ± standard deviation. Unpaired Student’s t-test, two- way ANOVA, *p<0.05, **p<0.01, ***p<0.001, ****p<0.0001.

**Supplementary Table 1. List of top 50 DE genes between control and Id depleted cells** The top 50 DE genes generated from the gene expression profile of three independent replicates of control and Id KD cells was compared by microarray analysis to generate a list of differentially expressed genes between Id depleted and control cells.

| ***S. No.*** | ***Gene.Symbol.x*** | ***Fold change*** | ***Direction*** | ***P Value*** | ***Q Value*** |
| --- | --- | --- | --- | --- | --- |
| 1 | *Mx2* :: myxovirus (influenza virus) resistance 2 | 26.6022 | up | 2.889E-12 | 7.591E-08 |
| 2 | *Oas1g* :: 2'-5' oligoadenylate synthetase 1G | 14.9575 | up | 7.929E-12 | 1.041E-07 |
| 3 | *Oas3* :: 2'-5' oligoadenylate synthetase 3 | 15.1238 | up | 2.227E-11 | 0.000000195 |
| 4 | *Cmpk2* :: cytidine monophosphate (UMP-CMP) kinase 2, mitochondrial | 24.3302 | up | 3.53E-11 | 2.318E-07 |
| 5 | *Stat1* :: signal transducer and activator of transcription 1 | 6.8185 | up | 6.255E-11 | 3.286E-07 |
| 6 | *Xaf1* :: XIAP associated factor 1 | 9.0698 | up | 9.273E-11 | 0.000000406 |
| 7 | *Usp18* :: ubiquitin specific peptidase 18 | 30.4897 | up | 1.111E-10 | 4.104E-07 |
| 8 | *Oas2* :: 2'-5' oligoadenylate synthetase 2 | 36.13 | up | 1.25E-10 | 4.104E-07 |
| 9 | *Ifit1*:: interferon-induced protein with tetratricopeptide repeats 1 | 18.532 | up | 1.567E-10 | 4.573E-07 |
| 10 | *Gpr56* :: G protein-coupled receptor 56 | 21.8354 | up | 1.817E-10 | 0.000000472 |
| 11 | *Zbp1* :: Z-DNA binding protein 1 | 13.5822 | up | 2.098E-10 | 0.000000472 |
| 12 | *Olfr65* :: olfactory receptor 65 | 8.3324 | up | 2.156E-10 | 0.000000472 |
| 13 | *Parp14* :: poly (ADP-ribose) polymerase family, member 14 | 5.9386 | up | 2.691E-10 | 5.218E-07 |
| 14 | *Angptl4* :: angiopoietin-like 4 | 12.1131 | up | 2.867E-10 | 5.218E-07 |
| 15 | *Irf7* :: interferon regulatory factor 7 | 13.4439 | up | 2.979E-10 | 5.218E-07 |
| 16 | *Gbp3* :: guanylate binding protein 3 | 10.1348 | up | 3.456E-10 | 5.674E-07 |
| 17 | *Stat2* :: signal transducer and activator of transcription 2 | 5.0425 | up | 4.398E-10 | 6.796E-07 |
| 18 | *Oasl2* :: 2'-5' oligoadenylate synthetase-like 2 | 5.7616 | up | 5.125E-10 | 7.454E-07 |
| 19 | *Bst2* :: bone marrow stromal cell antigen 2 | 8.43 | up | 5.396E-10 | 7.454E-07 |
| 20 | *Lypd3* :: Ly6/Plaur domain containing 3 | 4.2202 | up | 5.675E-10 | 7.454E-07 |
| 21 | *Iigp1* :: interferon inducible GTPase 1 | 14.3363 | up | 6.404E-10 | 7.932E-07 |
| 22 | *Pvrl1* :: poliovirus receptor-related 1 | 4.7013 | up | 6.643E-10 | 7.932E-07 |
| 23 | *Oas1b* :: 2'-5' oligoadenylate synthetase 1B | 9.7559 | up | 7.787E-10 | 8.894E-07 |
| 24 | *Megf10* :: multiple EGF-like-domains 10 | 11.0028 | up | 9.708E-10 | 0.000001044 |
| 25 | *Rtp4* :: receptor transporter protein 4 | 9.0025 | up | 9.932E-10 | 0.000001044 |
| 26 | *Dhx58* :: DEXH (Asp-Glu-X-His) box polypeptide 58 | 9.4499 | up | 1.133E-09 | 0.000001145 |
| 27 | *Irgm1* :: immunity-related GTPase family M member 1 | 3.8284 | up | 1.624E-09 | 0.00000158 |
| 28 | *Fst* :: follistatin | 6.2073 | up | 1.906E-09 | 0.000001788 |
| 29 | *Twf2* :: twinfilin, actin-binding protein, homolog 2 (Drosophila) | 3.1538 | up | 2.147E-09 | 0.000001931 |
| 30 | *Ifih1* :: interferon induced with helicase C domain 1 | 4.3871 | up | 2.215E-09 | 0.000001931 |
| 31 | *Gbp7* :: guanylate binding protein 7 | 7.9921 | up | 2.279E-09 | 0.000001931 |
| 32 | *17549062* | 3.6692 | up | 2.363E-09 | 0.000001939 |
| 33 | *BC006779* :: cDNA sequence BC006779 | 4.0514 | up | 2.523E-09 | 0.000002009 |
| 34 | *Eif2ak2* :: eukaryotic translation initiation factor 2-alpha kinase 2 | 3.4611 | up | 2.674E-09 | 0.000002066 |
| 35 | *Oas1a* :: 2'-5' oligoadenylate synthetase 1A | 8.1417 | up | 2.816E-09 | 0.000002114 |
| 36 | *Shf* :: Src homology 2 domain containing F | 2.7862 | up | 2.911E-09 | 0.000002124 |
| 37 | *Ecscr* :: endothelial cell surface expressed chemotaxis and apoptosis regulator | 4.0889 | up | 2.994E-09 | 0.000002126 |
| 38 | *Ifi44* :: interferon-induced protein 44 | 36.7437 | up | 3.392E-09 | 0.000002345 |
| 39 | *Gbp9* :: guanylate-binding protein 9 | 6.043 | up | 4.69E-09 | 0.000003159 |
| 40 | *Lcp1* :: lymphocyte cytosolic protein 1 | 3.8831 | up | 4.994E-09 | 0.00000328 |
| 41 | *Gstm5* :: glutathione S-transferase, mu 5 | 5.9088 | up | 5.454E-09 | 0.000003495 |
| 42 | 17549150 | 4.0644 | up | 5.665E-09 | 0.000003543 |
| 43 | *Sp100* :: nuclear antigen Sp100 | 6.721 | up | 5.864E-09 | 0.000003582 |
| 44 | *Igtp* :: interferon gamma induced GTPase | 14.6896 | up | 6.216E-09 | 0.000003639 |
| 45 | *Ubash3b* :: ubiquitin associated and SH3 domain containing, B | 3.4232 | up | 6.234E-09 | 0.000003639 |
| 46 | *Scube3* :: signal peptide, CUB domain, EGF-like 3 | 3.1924 | up | 6.474E-09 | 0.000003678 |
| 47 | *Cercam* :: cerebral endothelial cell adhesion molecule | 9.0449 | up | 6.753E-09 | 0.000003678 |
| 48 | *Cxcl11* :: chemokine (C-X-C motif) ligand 11 | 14.2065 | up | 6.962E-09 | 0.000003678 |
| 49 | *Ddx60* :: DEAD (Asp-Glu-Ala-Asp) box polypeptide 60 | 8.6685 | up | 6.964E-09 | 0.000003678 |
| 50 | *H19* :: H19 fetal liver mRNA | 2.8527 | down | 7.001E-09 | 0.000003678 |

**Supplementary Table 2.** **List of differentially expressed genes between the Id+ and Id- mouse TNBC cells generated from the Id1C3Tag model.**

| **S. No.** | **Gene name** | **logFC** | **P-value** | **Q-value** |
| --- | --- | --- | --- | --- |
| 1 | *Ibsp* | 34.70987 | 5.90E-32 | 1.49E-27 |
| 2 | *Car3* | 22.99498 | 2.63E-31 | 3.34E-27 |
| 3 | *Chad* | 29.2403 | 2.44E-22 | 2.06E-18 |
| 4 | *Alpl* | 13.80174 | 7.81E-18 | 4.95E-14 |
| 5 | *Fgg* | 0.016064 | 1.27E-13 | 6.43E-10 |
| 6 | *Wif1* | 11.50008 | 1.56E-13 | 6.60E-10 |
| 7 | *Upk1b* | 5.2646 | 1.24E-12 | 4.50E-09 |
| 8 | *Cyp1b1* | 3.053822 | 1.55E-10 | 4.91E-07 |
| 9 | *Comp* | 7.095195 | 1.70E-09 | 4.78E-06 |
| 10 | *Col8a2* | 5.33003 | 2.50E-09 | 6.33E-06 |
| 11 | *Thy1* | 0.072133 | 3.77E-09 | 7.34E-06 |
| 12 | *Daam2* | 5.753571 | 3.72E-09 | 7.34E-06 |
| 13 | *Oxtr* | 5.647227 | 3.68E-09 | 7.34E-06 |
| 14 | *Angptl4* | 0.364436 | 8.10E-09 | 1.47E-05 |
| 15 | *Bmp8a* | 35.35223 | 1.48E-08 | 2.50E-05 |
| 16 | *Cilp2* | 6.256408 | 2.79E-08 | 4.16E-05 |
| 17 | *Spink5* | 19.48619 | 2.73E-08 | 4.16E-05 |
| 18 | *Panx3* | 8.509771 | 4.80E-08 | 6.75E-05 |
| 19 | *Stc2* | 0.227554 | 6.35E-08 | 8.46E-05 |
| 20 | *Upp1* | 0.293682 | 2.00E-07 | 0.000254 |
| 21 | *C1qtnf3* | 0.17218 | 3.11E-07 | 0.000375 |
| 22 | *Aspm* | 1.943525 | 3.55E-07 | 0.000409 |
| 23 | *Gpr133* | 0.059733 | 4.17E-07 | 0.000459 |
| 24 | *Olfml2a* | 4.777964 | 7.71E-07 | 0.000814 |
| 25 | *Smad9* | 4.26899 | 8.80E-07 | 0.000892 |
| 26 | *BC106179* | 0.007113 | 1.01E-06 | 0.000986 |
| 27 | *Smoc2* | 3.83852 | 1.06E-06 | 0.00099 |
| 28 | *Zfhx4* | 0.027723 | 1.34E-06 | 0.00121 |
| 29 | *Id1* | 3.148029 | 1.48E-06 | 0.00129 |
| 30 | *Hmga1-rs1* | 0.472876 | 2.07E-06 | 0.001751 |
| 31 | *Cadm3* | 0.007485 | 2.45E-06 | 0.002 |
| 32 | *Angptl7* | 0.356076 | 2.63E-06 | 0.002085 |
| 33 | *Ltbp2* | 1.879608 | 2.78E-06 | 0.002135 |
| 34 | *Gas2l3* | 2.020165 | 2.93E-06 | 0.002182 |
| 35 | *Casz1* | 1.992817 | 3.08E-06 | 0.002231 |
| 36 | *Casc5* | 1.99469 | 3.22E-06 | 0.002269 |
| 37 | *Trim71* | 6.6267 | 3.50E-06 | 0.002394 |
| 38 | *3110079O15Rik* | 2.516533 | 3.65E-06 | 0.002435 |
| 39 | *Mylk* | 2.190019 | 5.24E-06 | 0.003408 |
| 40 | *Itga10* | 3.333803 | 6.08E-06 | 0.003853 |
| 41 | *Sulf1* | 0.155514 | 6.27E-06 | 0.003877 |
| 42 | *Cldn6* | 0.006279 | 7.02E-06 | 0.004138 |
| 43 | *Il6* | 2.423294 | 6.99E-06 | 0.004138 |
| 44 | *Ube2c* | 2.359489 | 8.44E-06 | 0.00486 |
| 45 | *Lox* | 0.252412 | 1.03E-05 | 0.005689 |
| 46 | *Fat2* | 3.213024 | 1.03E-05 | 0.005689 |
| 47 | *Grem2* | 0.008294 | 1.16E-05 | 0.006248 |
| 48 | *Scn7a* | 0.06646 | 1.32E-05 | 0.006944 |
| 49 | *Nusap1* | 2.131026 | 1.63E-05 | 0.008424 |
| 50 | *Ccnd1* | 0.48054 | 1.86E-05 | 0.00945 |
| 51 | *Chst13* | 6.796579 | 2.05E-05 | 0.010165 |
| 52 | *Cytl1* | 2.994287 | 2.62E-05 | 0.012746 |
| 53 | *Cxcl15* | 0.008277 | 2.77E-05 | 0.01323 |
| 54 | *Gfra2* | 0.328484 | 3.09E-05 | 0.014486 |
| 55 | *Ptx3* | 2.57852 | 3.23E-05 | 0.014626 |
| 56 | *Sfrp2* | 3.039032 | 3.23E-05 | 0.014626 |
| 57 | *Aurka* | 1.974289 | 3.54E-05 | 0.01575 |
| 58 | *Kif4* | 1.712909 | 3.89E-05 | 0.016983 |
| 59 | *Slpi* | 1.960889 | 3.98E-05 | 0.017109 |
| 60 | *Arhgap42* | 1.708361 | 4.67E-05 | 0.019716 |
| 61 | *Fgb* | 0.022514 | 4.81E-05 | 0.019924 |
| 62 | *Gm12324* | 6.61234 | 4.87E-05 | 0.019924 |
| 63 | *Aire* | 0.36178 | 5.06E-05 | 0.02035 |
| 64 | *Serpinb8* | 2.309948 | 5.28E-05 | 0.020453 |
| 65 | *Fam78b* | 0.007857 | 5.22E-05 | 0.020453 |
| 66 | *Cyp2b19* | 3.242025 | 5.33E-05 | 0.020453 |
| 67 | *Ctgf* | 2.026126 | 5.59E-05 | 0.020827 |
| 68 | *Prc1* | 1.714893 | 5.62E-05 | 0.020827 |
| 69 | *Mfap4* | 0.103597 | 5.67E-05 | 0.020827 |
| 70 | *Ptges* | 0.522245 | 5.77E-05 | 0.020886 |
| 71 | *Kif11* | 1.680348 | 6.37E-05 | 0.022222 |
| 72 | *Sdk2* | 2.449539 | 6.26E-05 | 0.022222 |
| 73 | *Sytl5* | 3.537568 | 6.40E-05 | 0.022222 |
| 74 | *Chil1* | 2.190271 | 6.58E-05 | 0.022522 |
| 75 | *Lef1* | 2.453506 | 7.00E-05 | 0.022795 |
| 76 | *Mmp3* | 0.458453 | 7.02E-05 | 0.022795 |
| 77 | *Fcgr3* | 0.013883 | 6.84E-05 | 0.022795 |
| 78 | *Wfdc3* | 3.703518 | 6.86E-05 | 0.022795 |
| 79 | *Tmeff2* | 3.820828 | 7.85E-05 | 0.025178 |
| 80 | *Chrdl2* | 4.840907 | 8.28E-05 | 0.026135 |
| 81 | *Dnm3* | 2.903093 | 8.42E-05 | 0.026135 |
| 82 | *Gramd2* | 2.748813 | 8.46E-05 | 0.026135 |
| 83 | *Cenpf* | 1.707497 | 8.98E-05 | 0.027428 |
| 84 | *Stmn2* | 3.600841 | 9.43E-05 | 0.028459 |
| 85 | *Frem1* | 4.723742 | 9.59E-05 | 0.028608 |
| 86 | *Lect1* | 4.066957 | 9.95E-05 | 0.029319 |
| 87 | *Sctr* | 16.04509 | 0.000102 | 0.029784 |
| 88 | *Tmem252* | 15.01066 | 0.000103 | 0.029793 |
| 89 | *Adam19* | 0.122191 | 0.000111 | 0.031557 |
| 90 | *Shc2* | 2.932148 | 0.000121 | 0.033596 |
| 91 | *Adamtsl3* | 2.531923 | 0.00012 | 0.033596 |
| 92 | *Pdgfc* | 1.733254 | 0.00013 | 0.035921 |
| 93 | *Peg3* | 2.447937 | 0.000141 | 0.037694 |
| 94 | *Syn1* | 2.457699 | 0.000141 | 0.037694 |
| 95 | *Sema3e* | 4.518764 | 0.000139 | 0.037694 |
| 96 | *Tmem56* | 0.28973 | 0.000149 | 0.039273 |
| 97 | *Zakit* | 5.98488 | 0.000156 | 0.040878 |
| 98 | *Plxdc2* | 2.138441 | 0.000162 | 0.041887 |
| 99 | *Scrg1* | 7.269112 | 0.00017 | 0.04347 |
| 100 | *Dsg1a* | 3.900087 | 0.00018 | 0.045529 |
| 101 | *Tshz3* | 0.016964 | 0.000183 | 0.045653 |
| 102 | *Loxl2* | 0.520959 | 0.000189 | 0.045653 |
| 103 | *Myo1d* | 1.681598 | 0.000186 | 0.045653 |
| 104 | *Ttc12* | 11.81049 | 0.000185 | 0.045653 |
| 105 | *Nhsl2* | 1.927395 | 0.000189 | 0.045653 |
| 106 | *Arsi* | 3.65595 | 0.000199 | 0.04762 |
| 107 | *Hus1b* | 0.016583 | 0.000207 | 0.048961 |
| 108 | *Lphn1* | 1.825075 | 0.000209 | 0.049079 |
| 109 | *Tmem47* | 3.039314 | 0.000211 | 0.049131 |
| 110 | *Lgr6* | 1.96859 | 0.000221 | 0.049982 |
| 111 | *Ctla2a* | 2.473585 | 0.000219 | 0.049982 |
| 112 | *Omp* | 2.821668 | 0.000218 | 0.049982 |

**Supplementary Table 3.** Candidate genes identified for further validation as putative Id target genes using siRNA screen.

| S. No. | miRNA.mimicsiRNA | Viability1 | Viability2 | Mean_Viability | Replicate_Disparity |
| --- | --- | --- | --- | --- | --- |
| 1 | *Actb* | 119.2385 | 69.44172 | 94.34012 | 0.199673 |
| 2 | *Adamtsl3* | 59.5834 | 72.47348 | 66.02844 | 0.051686 |
| 3 | *Alpl* | 90.50029 | 96.10138 | 93.30084 | 0.022459 |
| 4 | *ANGPTL4* | 47.11264 | 43.44723 | 45.27994 | 0.014697 |
| 5 | *Aspm* | 73.0994 | 49.45445 | 61.27693 | 0.094811 |
| 6 | *AURKA* | 47.13871 | 62.01435 | 54.57653 | 0.059648 |
| 7 | *Axin2* | 120.1843 | 168.8596 | 144.522 | 0.195177 |
| 8 | *BMI1* | 76.35547 | 93.35345 | 84.85446 | 0.068158 |
| 9 | *BMP8A* | 54.38554 | 78.88393 | 66.63473 | 0.098233 |
| 10 | *Casc5* | 12.91458 | 20.8636 | 16.88909 | 0.031874 |
| 11 | *CCND1* | 2.362772 | 2.487312 | 2.425042 | 0.000499 |
| 12 | *Ccne1* | 107.1427 | 101.5924 | 104.3675 | 0.022255 |
| 13 | *Cdkn1a* | 151.815 | 187.5555 | 169.6852 | 0.143311 |
| 14 | *Cdkn2a* | 9.258506 | 12.23492 | 10.74671 | 0.011935 |
| 15 | *CENPF* | 104.4639 | 126.2672 | 115.3655 | 0.087426 |
| 16 | *CHAD* | 161.2562 | 192.6356 | 176.9459 | 0.125824 |
| 17 | *CLDN6* | 5.437018 | 16.92079 | 11.1789 | 0.046047 |
| 18 | *Col8a2* | 92.66478 | 91.96999 | 92.31739 | 0.002786 |
| 19 | *Comp* | 112.4995 | 166.9346 | 139.717 | 0.218272 |
| 20 | *CXCL15* | 11.52211 | 22.00924 | 16.76568 | 0.042051 |
| 21 | *Dpysl2* | 83.76739 | 73.22459 | 78.49599 | 0.042274 |
| 22 | *FOXC2* | 32.42075 | 51.92788 | 42.17431 | 0.078219 |
| 23 | *GAPDH* | 74.66308 | 129.214 | 101.9385 | 0.218736 |
| 24 | *Gp9* | 67.24151 | 72.35828 | 69.79989 | 0.020517 |
| 25 | *GPR133* | 66.20689 | 72.52594 | 69.36641 | 0.025338 |
| 26 | *GREM2* | 14.9732 | 27.7484 | 21.3608 | 0.051226 |
| 27 | *Gypa* | 55.29883 | 41.61613 | 48.45748 | 0.054864 |
| 28 | *Ibsp* | 56.76146 | 76.95146 | 66.85646 | 0.080957 |
| 29 | *ID1* | 119.4619 | 135.7036 | 127.5827 | 0.065126 |
| 30 | *ID2* | 25.04487 | 48.90737 | 36.97612 | 0.095683 |
| 31 | *ID3* | 26.8322 | 26.7894 | 26.8108 | 0.000172 |
| 32 | *Id4* | 60.58005 | 61.8837 | 61.23187 | 0.005227 |
| 33 | *Il6* | 54.41804 | 38.86627 | 46.64216 | 0.062359 |
| 34 | *Kif11* | 5.929065 | 10.06528 | 7.997174 | 0.016585 |
| 35 | *Kif4* | 137.4777 | 145.0792 | 141.2785 | 0.03048 |
| 36 | *Lef1* | 69.74422 | 75.35658 | 72.5504 | 0.022504 |
| 37 | *Lgr6* | 13.77349 | 27.60068 | 20.68709 | 0.055444 |
| 38 | *Lox* | 30.49407 | 53.24569 | 41.86988 | 0.091229 |
| 39 | *Loxl2* | 33.93647 | 41.46456 | 37.70052 | 0.030186 |
| 40 | *Myom1* | 78.83341 | 66.06561 | 72.44951 | 0.051196 |
| 41 | *Nusap1* | 26.12422 | 18.19966 | 22.16194 | 0.031776 |
| 42 | *Oxtr* | 14.45927 | 29.72044 | 22.08986 | 0.061194 |
| 43 | *Panx3* | 70.37883 | 95.81948 | 83.09915 | 0.102011 |
| 44 | *Pdgfc* | 48.00148 | 46.44553 | 47.2235 | 0.006239 |
| 45 | *POSTN* | 57.7375 | 78.37966 | 68.05858 | 0.08277 |
| 46 | *Prc1* | 44.28233 | 36.23709 | 40.25971 | 0.03226 |
| 47 | *ROBO1* | 249.3912 | 225.3732 | 237.3822 | 0.096306 |
| 48 | *Scrn1* | 219.7686 | 218.585 | 219.1768 | 0.004746 |
| 49 | *Sctr* | 102.5398 | 85.73719 | 94.13851 | 0.067375 |
| 50 | *Sfrp2* | 113.2776 | 131.8863 | 122.582 | 0.074616 |
| 51 | *Smad9* | 37.48249 | 43.25865 | 40.37057 | 0.023161 |
| 52 | *Spaca3* | 46.28752 | 72.89763 | 59.59258 | 0.1067 |
| 53 | *Stom* | 174.221 | 165.451 | 169.836 | 0.035165 |
| 54 | *TGFBR3* | 123.9611 | 98.65301 | 111.307 | 0.101479 |
| 55 | *Tmem252* | 9.216349 | 8.13346 | 8.674905 | 0.004342 |
| 56 | *Tmem47* | 33.99214 | 59.51196 | 46.75205 | 0.102328 |
| 57 | *Ube2c* | 66.20978 | 93.01105 | 79.61042 | 0.107467 |
| 58 | *VCAM1* | 20.82949 | 33.1387 | 26.9841 | 0.049357 |
| 59 | *Vps51* | 46.86967 | 34.84365 | 40.85666 | 0.048222 |
| 60 | *WIF1* | 31.32273 | 52.26417 | 41.79345 | 0.08397 |
| 61 | *ylk* | 103.3257 | 62.85685 | 83.09127 | 0.162271 |

**Supplementary Table 4.** List of quantitative real time PCR primers used in the study.

| **Oligo name** | **Sequence (5’-3’)** |
| --- | --- |
| Casc5-F#1  Casc5-R#1 | TCGCTGAAGTGGAAACAGAAAC  TATCTGAGCAAGGGTCTGCG |
| Rb-F#1  Rb-R#1 | CATCCTTTAACTCTAAGGCCTCATTC  CAGAAGGCGTGCACAGAGTGT |
| Kif11-F#1  Kif11-R#1 | GAGGAAAAGGGCAGGAACAT  CTTTCCGCTCTGCCAGATTA |
| CyclinA2-F#1  CyclinA2-R#1 | CAAGACTCGACGGGTTGCTC  GCTGGCCTCTTCTGAGTCTC |
| CyclinB1-F#1  CyclinB1-R#1 | GGTGTAACGGCCATGTTTATTG  CTGTCTGATCTGGTGCTTAGTG |
| p21-F#1  p21-R#1 | GCAGATCCACAGCGATATCC  CAACTGCTCACTGTCCACGG |
| CyclinE1-F#1  CyclinE1-R#1 | CTGAGAGATGAGCACTTTCTG  CTGAGAGATGAGCACTTTCTG |
| p16-F#1  p16-R#1 | GAACTCTTTCGGTCGTACCC  CGAATCTGCACCGTAGTTGA |
| CDK4-F#1  CDK4-R#1 | TATGAACCCGTGGCTGAAAT  CCTTGATGTCCCGATCAGTT |
| CDK6-F#1  CDK6-R#1 | GCCTATGGGAAGGTGTTCAA  GGGCTCTGGAACTTTATCCA |
| CDK1-F#1  CDK1-R#1 | TTTAGGTTTGTTGTAAAGC  TAAGAGAGGACAGGAGATT |
| CDK2-F#1  CDK2-R#1 | TCCTCTGAGAGCAGTGATGCA  TTCCCCCAATGACCTAACCAG |
| p53-F#1  p53-R#1 | AAGATCCGCGGGCGTAA  CATCCTTTAACTCTAAGGCCTCATTC |
| Aurka-F#1  Aurka-R#1 | CACACGTACCAGGAGACTTACAGA  AGTCTTGAAATGAGGTCCCTGGCT |
| β-actin-F#1  β-actin-R#1 | AAGGCCAACCGTGAAAAGAT  GTGGTACGACCAGAGGCATAC |
| Ccnd1-F#1  Ccnd1-R#1 | GCCGAGAAGTTGTGCATCTA  TCACCAGAAGCAGTTCCATTT |
| Id1-F#2  Id1-R#2 | CCTAGCTGTTCGCTGAAGGC  GTAGAGCAGGACGTTCACCT |
| Id3-F#2  Id3-R#2 | CTGTCGGAACGTAGCCTGG  GTGGTTCATGTCGTCCAAGAG |

**Supplementary Table 5.** List of western blot antibodies used in the study.

| **Antibody** | **Catalogue Number** | **Secondary Antibody** | **Protein loaded** | **Dilution of primary antibody** | **Storage** | **Molecular weight** |
| --- | --- | --- | --- | --- | --- | --- |
| Casc-5  (Polyclonal) | Ab70537 | Anti-rabbit | 20 ug | 1:1000 | -20 | 250kDa |
| Kif-11  (Polyclonal) | HPA010568 | Anti-rabbit | 20ug | 1:500 | -20 | 119kDa |
| Aurora Kinase | SAB4502886 | Anti-rabbit | 20ug | 1:500 | -20 | 48kDa |
| Cyclin D1  (mAb)  (92G2) | 2978S | Anti-rabbit | 20ug | 1:1000 | -20 | 37kDa |
| Id1 | Biocheck | Anti-rabbit | 20ug | 1:500 | -20 | 17kDa |
| β- actin | sigma | Anti mouse | 20ug | 1:10000 |  | 42kDa |
