# Supplementary figures and images for: Targeting the Id1-Kif11 Axis in Triple-Negative Breast Cancer Using Combination Therapy
Source: Biomolecules. 2020 Sep 8;10(9):1295. doi: 10.3390/biom10091295 (PMC7565337; doi:10.3390/biom10091295)

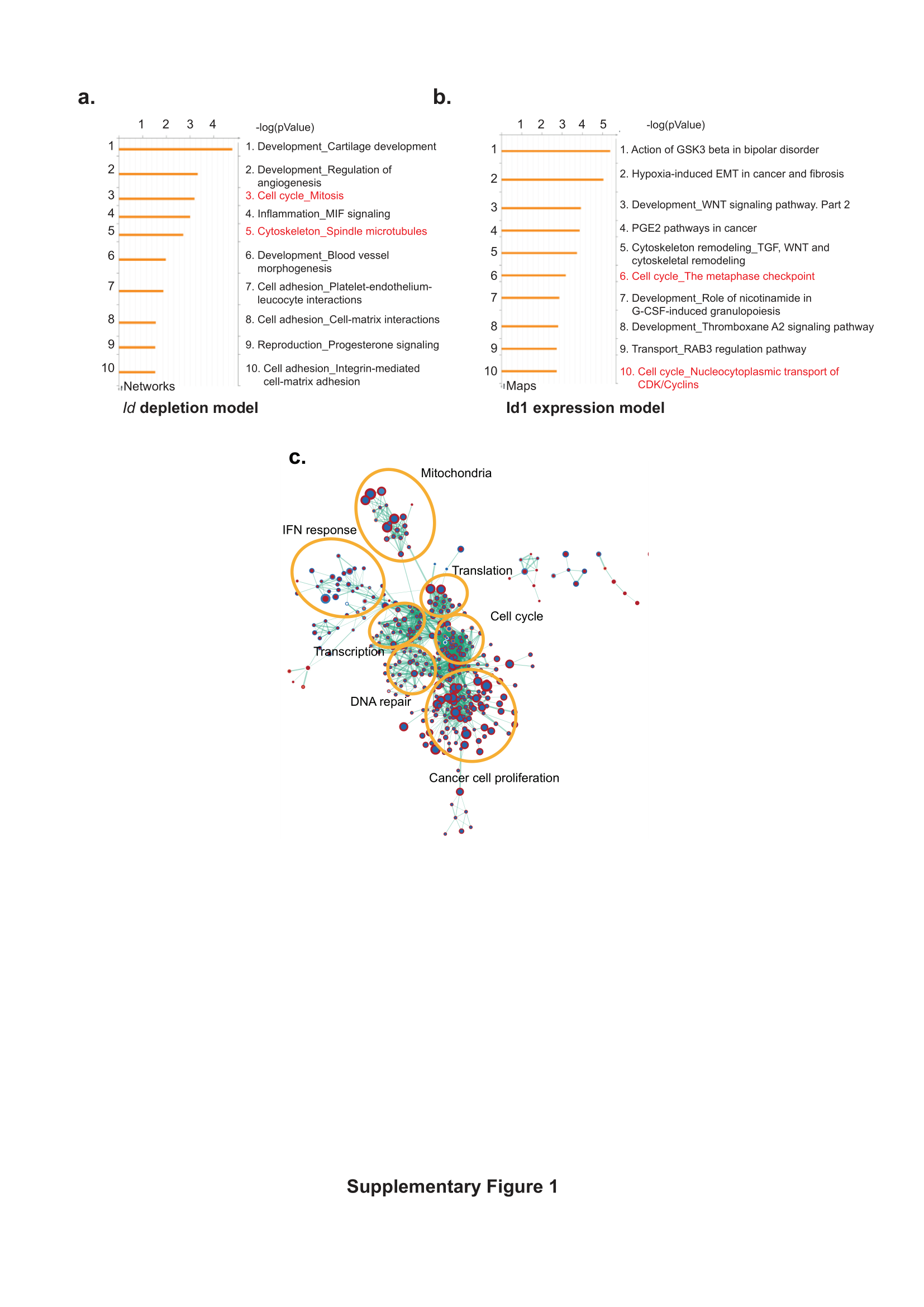

Supplement: Supplementary file 1 [file biomolecules-10-01295-s001.zip › supplementary files/Supplementary figures/20200814 Supplementary Figure 1 final.tiff]

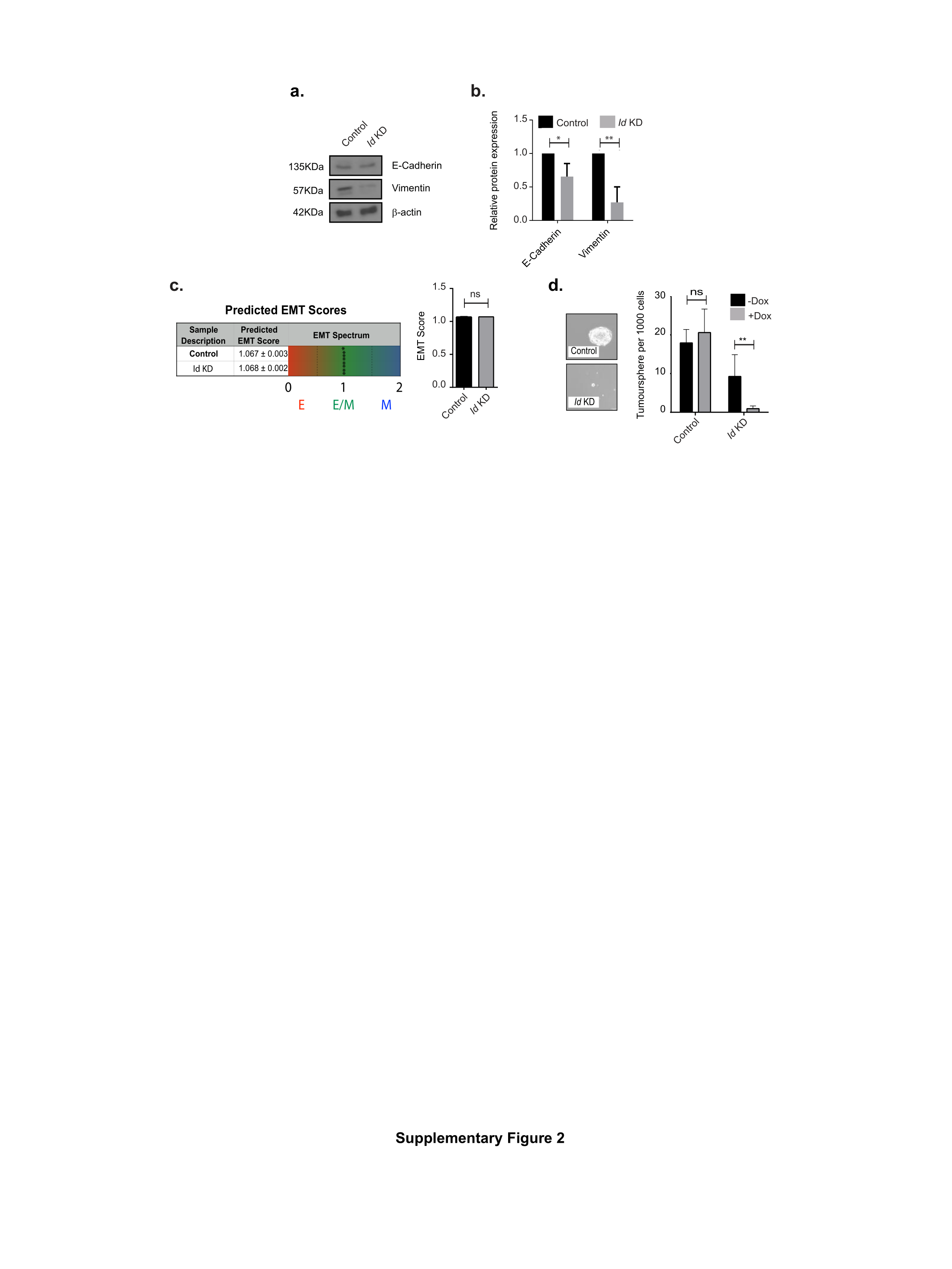

Supplement: Supplementary file 1 [file biomolecules-10-01295-s001.zip › supplementary files/Supplementary figures/20200814 Supplementary Figure 2 final.tiff]

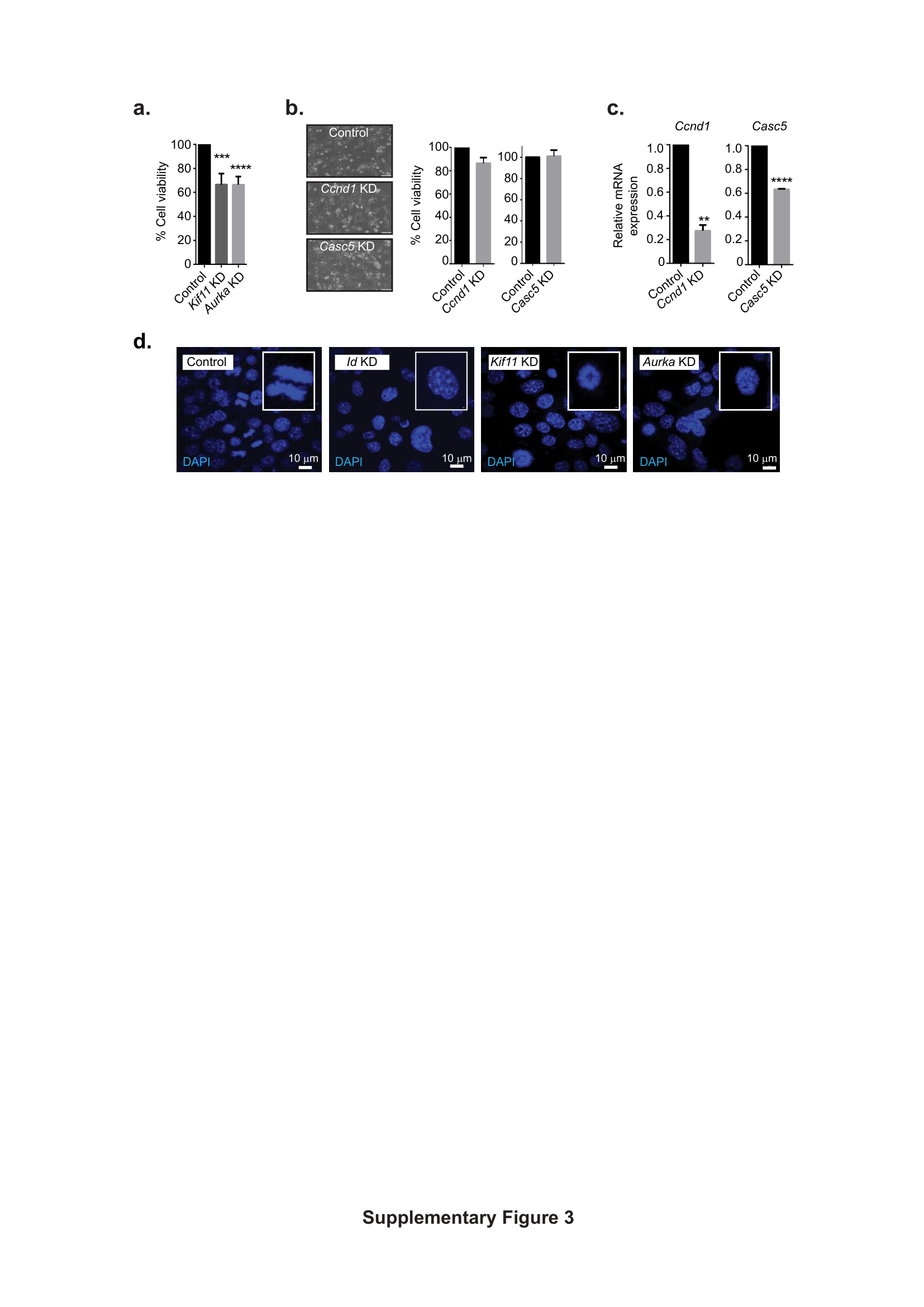

Supplement: Supplementary file 1 [file biomolecules-10-01295-s001.zip › supplementary files/Supplementary figures/20200814 Supplementary Figure 3 final.tiff]

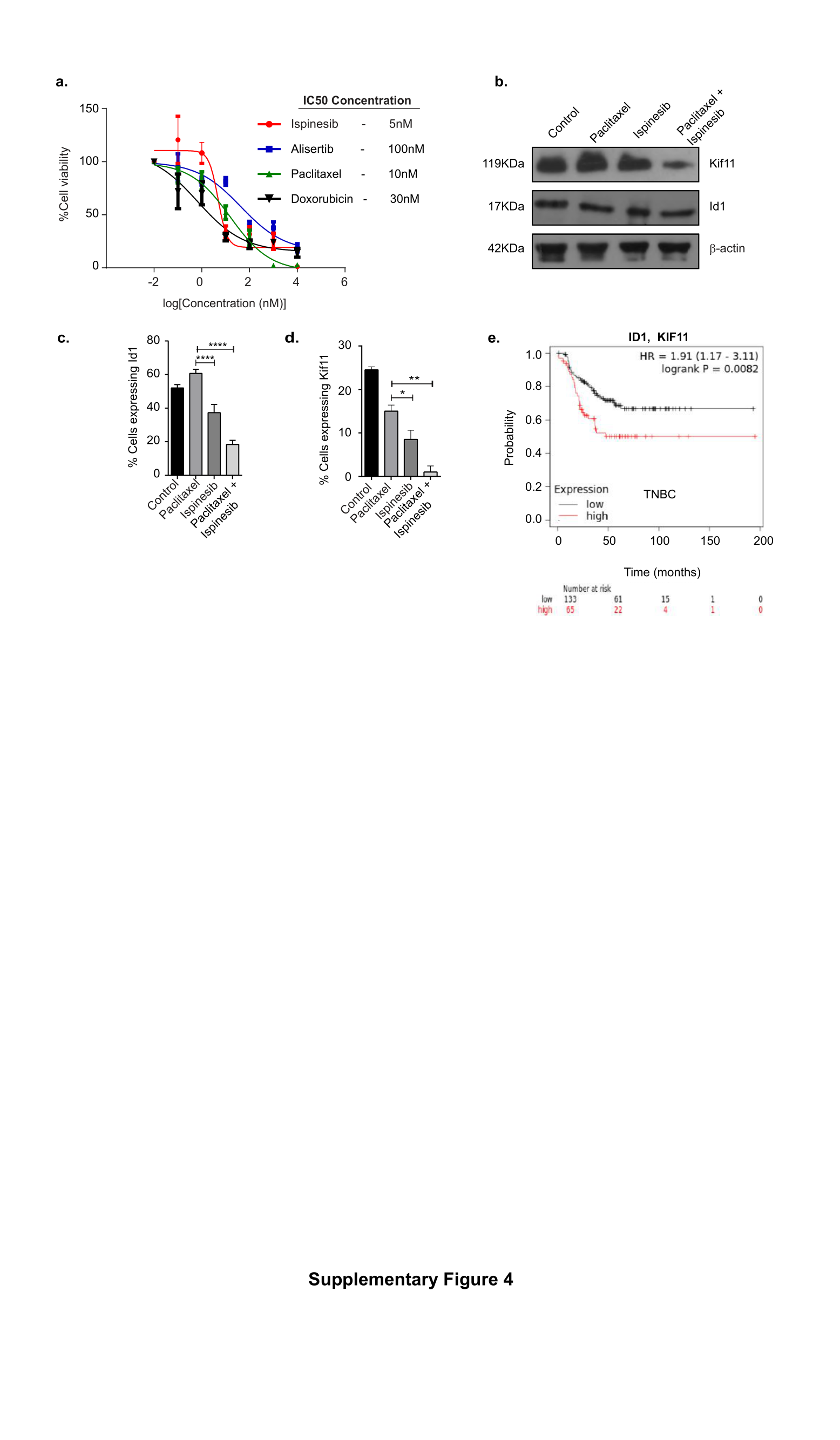

Supplement: Supplementary file 1 [file biomolecules-10-01295-s001.zip › supplementary files/Supplementary figures/20200814 Supplementary Figure 4 final.tiff]
